# Transforming the interrelated nature of human psychoneuroendocrine health and endocrine disrupting compounds in our planet’s water: from Wilhelm Waldeyer’s neuron theory to an artificial intelligence extension of the human body?

**DOI:** 10.3389/fmed.2025.1583203

**Published:** 2025-09-15

**Authors:** Sophie Schweizer-Schubert, Götz von Waldeyer-Hartz, Susann-Elisabeth Schütze, Daniel Mahringer, Aki Sebastian Ruhl, Markus Graf, Jochen Kuckelkorn

**Affiliations:** ^1^International Psychoendocrinology and Psychotherapy Practice, Heilbronn, Germany; ^2^Institute of Medical Psychology, Heidelberg University Hospital, Heidelberg, Germany; ^3^Adeamus CC Fluid Systems Research and Technology, Heilbronn, Germany; ^4^Section of Toxicology of Drinking Water and Swimming Pool Water, German Environment Agency (UBA), Bad Elster, Germany; ^5^Section of Water Treatment, German Environment Agency (UBA), Berlin, Germany; ^6^Technische Universität Berlin, Water Treatment, Berlin, Germany; ^7^Faculty of Informatics, School of Applied Artificial Intelligence, Heilbronn University of Applied Sciences, Heilbronn, Germany

**Keywords:** endocrine disrupting compounds, psychoneuroendocrinology, mental health, stress-related disorders, human health, drinking water, wearable saliva sensor, artificial intelligence

## Abstract

The interplay between steroid hormones (reproductive and stress hormones) and mental and physical health has evolved as an important area of medical and psychological research. At the same time, endocrine disrupting compounds (EDCs) spreading via our planet’s water have become a focus in environment- and health-related sciences, as well as in the public interest. The impact of EDCs on the delicate hormonal balance essential to human health remains insufficiently understood. The Federal Ministry of Health in Germany deemed this topic so important that it tasked the German Environment Agency with conducting a nationwide, effect-directed analysis of EDCs in drinking water. Our interdisciplinary research collaboration, providing its scientific foundation, includes expertise from medicine, psychology, biology, ecotoxicology, technology, and artificial intelligence. The objective of this review is the assessment of endocrine effects caused by drinking water on the human body and the reduction of EDCs in the urban water cycle emitted by the human body. Our specific goals are to gain a better understanding of human psychoneuroendocrine health in relation to the EDC problem, to identify gaps in current research and to explore measures for reducing the human body’s emissions of EDCs. This assessment is particularly relevant given the anticipated global rise in the use of contraceptives, infertility treatments, hormone-replacement therapies and endocrinological treatments of stress-related disorders, all of which contribute to increased endocrine-disrupting compounds in the water cycle. Leveraging artificial intelligence and virtual human twin technologies to simulate individualized hormonal responses provide valuable insights into possible targeted interventions for reducing EDCs by personalized endocrinological practice.

## 1 Introduction

Endocrine disrupting compounds (EDCs) spreading via our water and accumulating in various systems are increasingly becoming a focus in health and environment-related sciences, as well as public interest. The European Commission provides a comprehensive definition: “An endocrine disrupter is an exogenous substance or mixture that alters function(s) of the endocrine system and consequently causes adverse health effects in an intact organism, or its progeny, or (sub)populations.” (1). The substances so far identified as EDCs are highly heterogenous and comprised of natural chemicals, e.g., phytosteroids, as well as synthetic chemicals, e.g., 17α-ethinylestradiol, atrazine, bisphenol A, phthalates, polychlorinated biphenyls, or dioxins. Known sources of synthetic EDCs are pesticides, pharmaceuticals, personal care products, industrial solvents/lubricants, plasticizers, and flame retardants (2). The specific impacts of EDCs present in our water, including drinking water, on the delicate hormonal balance essential to human health have not yet been sufficiently clarified. Many metabolic processes are controlled by the endocrine system, i.e., regulation of homeostasis, reproduction, as well as the development and behavior of human beings. Examples of hypothesized adverse effects of EDCs on human health include feminization of male gonads (3), neurodegenerative diseases (4), thyroid disfunction (5), reduced fertility, diabetes, obesity, carcinogenesis (6) and even mental health, as in the case of attention deficit and hyperactivity disorder (ADHD) (7). Comprehensive reviews of the scientific literature on possible impacts of EDCs on human health were provided by Colburn et al. as early as 1997 (8), and more recently by Kabir et al. (9). Vulnerabilities and risk factors in human health, as well as in the reproductive hormonal system have been explicated for EDCs.

At the same time, the interplay between steroid hormones (reproductive hormones and stress hormones) and mental and physical health has evolved as an important area of medical and psychological research [e.g., (10–12)]. The consequences of EDC emissions into the urban water cycle and endocrine effects of EDCs on hormone-related disorders involving stress hormones and reproductive hormones will need to become a key part of the EDC discussion. This is the group of disorders in which one would expect endocrine disruption to be most relevant. An entire Frontiers Research Topic was dedicated to psychoneuroendocrine health (13, 14). Prominent examples of psychoneuroendocrine disorders belonging to this diagnostic category are stress-related disorders, such as what is colloquially referred to as “burnout,” or disorders in reproductive transitions like perimenopausal depression, premenstrual affective disorders, postpartum depression and depression during puberty.

The interference of EDCs with human health can be caused by different modes of action in our endocrine system. EDCs can bind to endocrine receptors where they behave as “hormone mimics,” keeping natural hormones from working within the human body as they naturally do; other EDCs bind to endogenous hormones, disrupt enzymes and co-factors of the steroidogenesis or transport proteins of the endocrine system. Receptors might be activated or blocked, and hormones, enzymes, co-factors and transport proteins might be enhanced or impaired in their function, leading to altered hormone levels in target tissues, and ultimately resulting in altered physiological responses (15). But what does our drinking water really do at human endocrine receptors? The Federal Ministry of Health in Germany (in German: *Bundesministerium für Gesundheit*, BMG) deemed the topic of EDCs to be important enough to commission a nationwide, effect-directed analysis of German drinking water to the German Environment Agency (in German: *Umweltbundesamt*, UBA). The topic of EDCs in the environment received considerable attention following an international conference in Weybridge, England (16). The European Commission has officially worked on the topic of EDCs since the turn of the millennium (1, 17).

Currently the Danish Environmental Protection Agency administers a website on which three Endocrine Disruptor lists are made public. The first of these lists substances identified as endocrine disruptors at EU level, the second one lists substances under evaluation for endocrine disruption under an EU legislation and the third lists substances considered by the evaluating national authority, to have endocrine disrupting properties. This website is a collaboration of the national authorities of Belgium, Denmark, France, The Netherlands, Sweden and Spain to provide information about regulatory status of EDCs (18).

The ubiquitous and all-encompassing reach of micropollutants can be understood when considering persistent and mobile chemicals such as Perfluoroalkyl and Polyfluoroalkyl Substances (best known by their abbreviation PFAS), as well as their metabolite, trifluoracetic acid (TFA). These substances are suspected of causing numerous adverse effects, such as those on the thyroid (19, 20), and are now found almost all over the world, even in completely uninhabited regions (21–23). EDCs enter the urban water cycle via different pathways and can be detected in the marine environment, surface waters, groundwater and - in trace concentrations - drinking water (24–28). Substances migrating from materials in residential plumbing installations, such as plasticizers in pipes, can also affect the hormone system. Many organic materials in contact with our drinking water could potentially release substances that cause endocrine disruption. EDCs enter the human body through the food chain (29). A common pathway is the consumption of fish, which could reflect various accumulation processes. For example, bioconcentration occurs when fish absorb substances such as Bisphenol A (BPA) directly from water through the gills and skin. Bioaccumulation refers to the uptake of EDCs from all environmental sources, including water, food and sediment. Finally, biomagnification is the increase in concentration of EDCs on their way up the food chain – such as when humans eat fish that have consumed smaller organisms already contaminated with EDCs (30). Other potential routes of EDCs into the human body are inhalation, for instance via fragranced personal care products evaporated into the air, as well as dermal absorption (31).

While the human body has its own mechanisms for removing xenobiotic compounds by means of the xenobiotic metabolism, this does not provide perfect health protection. Xenobiotics are defined as chemical compounds that are present in, but foreign to, biologic systems (32). Xenobiotics may be deactivated by the liver, and excreted via urine, feces, breath or sweat. However, another possibility is that xenobiotics are converted into more toxic forms through a process of bioactivation that, for example, can change the microbiome structurally and functionally (33). The link between the state of an individual’s microbiome and mental health has been well established (34). Given this fact, the discussion of moderators of mental health affecting human endocrinology, in particular psychoneuroendocrine disorders related to stress and reproductive hormonal mechanisms (14, 35), might need to be expanded to include environmental influences such as EDCs potentially occurring in water, which have not yet been considered.

As far as EDCs in drinking water are concerned, studies are scarce or missing. Therefore, research on the actual effect of potential EDCs in drinking water is needed. The question as to whether human exposure to EDCs is greater through drinking water or through food remains unanswered. In addition, it is still unclear what the main EDCs in drinking water are. Natural waters including marine and surface waters are contaminated by residual waters from all anthropogenic sources, be they domestic, from hospitals, from agriculture or industry. Policy makers need to identify at which point in the urban water cycle the most urgent action is required in order to reduce EDCs in water. This holds true not only when it comes to the treatment of EDCs in water, but also with respect to their release in the first place. As a consequence of progress in chemical synthesis and the vast application of chemicals in many fields of human life, EDCs can be found in many compartments of the aquatic environment. Established treatment processes such as ozonation, membrane filtration, and adsorption onto activated carbon or other adsorbents remove numerous organic micropollutants (including EDCs) and effects in bio-assays by advanced wastewater treatment (36). Due to the diversity of EDC predictions, respective removals are difficult and experimental studies are required. Direct measurement of endocrine effects with bio-assays might be a good approach for the evaluation of treatment methods for EDC removal if the concentrations are sufficiently high to show effects.

One of the contributions of this review is to follow-up on the previous Frontiers research topic on psychoneuroendocrine health, by associating the topic of endocrine disruption via water with psychoneuroendocrine disorders. This review primarily constitutes the foundation of an interdisciplinary collaboration of researchers in medicine, psychology, biology, (eco-)toxicology and technology, as well as Artificial Intelligence (AI). The two main objectives of this review are, on the one hand, the assessment of endocrine effects on the human body caused in part by drinking water and, on the other hand, the reduction of EDCs released into the urban water cycle caused by emissions from the human body. Our goals are to gain a better understanding of human psychoneuroendocrine health in relation to the EDC problem while identifying humans as a hereto forth neglected EDC emitting unit, identifying gaps in the current research and proposing measures to mitigate EDC emissions through the use of AI. We consolidate our insights for a better understanding of this challenge faced by humans, as well as the increasing health anxiety among the world’s population in the face of the EDC discourse. Such concerns must be addressed through novel scientific research and the problem needs to be solved by evidence-based action.

In this review we present current approaches to evaluating and reducing human exposure to EDCs and identify one crucial gap: In the effort to reduce the release of EDCs in the first place, there needs to be a sharper focus on the human body and its role in emitting EDCs into the urban water cycle, especially by current medical practice. There is a pronounced lack of ideas for practical solutions to be transformed into feasible plans for reducing human emissions of EDCs. A derailing human species with a mental health pandemic on the rise might be a dystopic scenario, the beginning of which we might witness in our time. How can innovations focusing on the human body and health, in terms of emission reduction, play an integral part in health promotion and prevention in the face of EDCs? We argue that AI as an extension to the human psychoneuroendocrine system may be a key field, not only in the context of reducing human emissions currently exacerbating the EDC problem, but also in the establishment of properly dosed and efficiency-controlled pharmacological interventions to avoid the excretion of excesses into our water and unnecessary burdens on the human body itself.

## 2 How the German Environment Agency (UBA) seeks to understand the effect of endocrine disrupting compounds in our water on human endocrine receptors

Currently, German drinking water regulation covers EDCs as micropollutants indirectly via the general precautionary value of 0.1 μg/l. When this value is exceeded by a substance, an evaluation of the substance must follow (37). Since toxicological data is scarce or missing for many upcoming micropollutants, traditional toxicological evaluation is often not possible. In the joint project “Tox-Box,” the German Environment Agency UBA therefore developed a guideline to address the potential hazard of these substances from an effect-oriented point of view, looking at the modes of action geno-, neuro-, reproductive and immunotoxicity, as well as endocrine disruption (38, 39). Working with the concept of a health-related indicator value (HRIV, in German: *Gesundheitlicher Orientierungswert*, GOW) micropollutants are to be assessed with suggested test systems and an HRIV as a trigger value in [μg/l] is then derived to be used for regulatory decisions. The established concept follows a precautionary principle to protect human health (40).

For endocrine disruption, the HRIV for any substance showing an effect on the estrogen receptor 17α is 0.01 μg/l relative to the 17beta-estradiol equivalent concentration (41). This is 10× less than the precautionary value of 0.1 μg/l, leaving room for substances to be present in concentrations of concern without being addressed. Due to the existing regulations (for single substances) and taking into account possible mixture effects, it is important to examine how a reliable assessment of the endocrine activity of (drinking) water can be made, based on the current data in Germany and Europe. The State Institute for Chemical and Veterinary Analysis of Food in Stuttgart (CVUA) reported up to a maximum of 211 μg/l BPA in warm water samples from household installations with epoxy linings (42), eighty times higher than the current limit (43). BPA is a known EDC, showing carcinogenic and reproductive effects (44, 45). However, high BPA concentrations were not found in cold water samples, suggesting that increased water temperatures accelerate the migration of EDCs from inappropriate materials.

**FIGURE 1:**
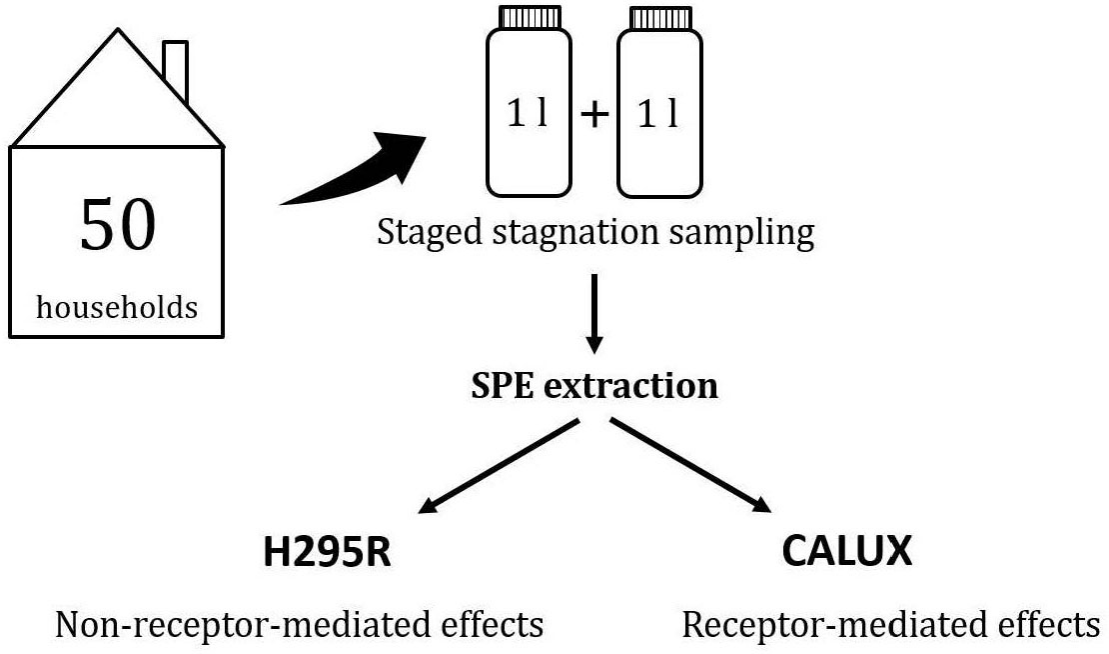
Outline of the study “monitoring of endocrine activity in drinking water in Germany” (120).

The project HoWiTri (Monitoring of endocrine activity in drinking water in Germany), funded by the Federal Ministry of Health in Germany, aims at closing the above mentioned data gap by evaluating potential effects of 50 different tap waters on human endocrine receptors (46). The sampling locations cover different degrees of urbanization (rural versus urban), different sources of raw water (groundwater versus surface water), and different ages of respective drinking water plumbing installations in houses (less or more than 5 years). Samples were extracted and analyzed with CALUX^®^ assays for receptor-mediated effects (estrogen receptor 17α, thyroid receptor 17β, glucocorticoid receptor, androgen receptor and progesterone receptor) and with the H295R steroidogenesis assay (47, 48) for non-receptor-mediated effects (41). Figure 1 provides an overview of the structure of the ongoing study.

## 3 How psychoneuroendocrine disorders and their treatment are associated with endocrine disrupting compounds in the urban water cycle

Navigating the psychoneuroendocrine health of human beings between hormonal and environmental factors of an individual has recently come into focus in the international psychoneuroendocrinological research community (14). Environmental factors associated with individuals, such as social factors (e.g., social stressors), were researched in their interaction with hormonal factors. For women, the interplay between the reproductive hormone system (gonadal hormones) and the stress hormone system in mental health has become recognized as a highly relevant issue and has been researched in particular depth (12). These endocrine mechanisms are particularly vulnerable during reproductive transitions such as menarche, menstrual cycle, peripartum and menopause. A vast amount of scientific evidence has shown that fluctuations in reproductive hormones likely account for a significant proportion of depressive symptoms. Men also represent a fair share of patients with psychoneuroendocrine disorders when treated for e.g., what is colloquially referred to as “burnout.” Stress-related hormonal disorders are increasingly treated by hormonal interventions (11). Cortisol levels or adrenal diseases need to be monitored in this context. However, what role EDCs may play in this group of psychoneuroendocrine disorders has not yet been clarified, which includes on the one hand the effect of the pharmacological treatments of these disorders as an EDCs-emitting factor burdening our water, as well as the subsequent effect of EDCs on these disorders as such, when the water reaches the human body again. The possible link between EDCs and the stress hormonal system has been discussed in a review by Kabir et al. (9). Much earlier, the association between mental illness and stress was well established and is known as the stress vulnerability model (49). Also highly relevant are possible effects on the thyroid, the optimal functioning of which is also crucial to mental health. PFAS have been argued to cause effects on the thyroid (19, 20).

In order to illustrate how delicate balances can become in human endocrine transition periods and how easily they can become disordered, we provide here some examples: Sex differentiation in the prevalence of depression begins with hormonal changes at puberty (50). Before puberty, depression rates are similar in girls and boys, but slightly higher in boys (51). After menarche (the first menstruation) the prevalence of mood disorders and suicidal behavior rises sharply in women (52). EDCs have indeed been argued to influence the onset of puberty (53); another example: it has been shown that female hormonal fluctuations are closely related to the male partners’ hormonal fluctuations. Testosterone decline in the male partner prior to childbirth reduces aggression and increases social support for the female partner. Findings suggest an increased risk for female perinatal depression when these co-fluctuations are out of sync in a couple (54). EDCs could bring such delicate balances out of sync. Similar influences are conceivable in premenstrual mood disorders, another sub-group of reproductive mood disorders, that is also well-researched (55). It is estimated that 13%–19% of naturally cycling women experience clinically significant depressive symptoms in the premenstrual phase (56, 57). With respect to another reproductive transition, in the DSM-5 (58), there is a “peripartum onset” specifier that includes both pregnancy and postpartum in the affective disorders section, illustrating how prevalent and relevant depression is in the peripartum. Up to 25% of women experience significant depressive symptoms following pregnancy (59, 60). Stress-inducing factors for depression and anxiety, for instance, during pregnancy, have been shown to be risk factors for these disorders (61); and finally: the perimenopausal transition, usually between the ages of 42 and 55, is also associated with elevated depressive symptoms given the conclusion that 45%–68% of perimenopausal women, versus only 28%–31% of premenopausal women, report such clinically significant elevations in depressive symptoms (62). Several studies have shown that fluctuation in estradiol (E2) plays a key role in the development of perimenopausal depression (63). Endocrinological as well as psychopharmaceutical treatments for these kinds of human disorders constitute one of the origins of EDCs in water. In turn, the interplay between our hormones and EDCs potentially migrating into warm water (as a non-social environmental factor) may disturb delicate endocrine processes, which may result in a vicious circle perpetuated through the urban water cycle. Researchers still need to specify to what extent these mechanisms may be active already, what the EDCs and main problematic prescription drugs are, as well as which of those are found in residual, surface and drinking waters.

The relationship between pharmaceuticals and human mental health has been a troubled one, and the same is true for the relationship between pharmaceuticals and our water. Impressive scientific findings show that synthetic contraceptive hormones are associated with a significant prevalence of depressive symptoms for those who take them (64). Things look very different for the medical use of hormone replacement therapy (HRT), particularly the body-identical HRT which is chemically identical to the hormones in the female body. It is made from plants and is strictly regulated, meeting the safety requirements of FDA-approved drugs produced by pharmaceutical companies. This is to be distinguished from the unregulated compounded bioidentical hormone therapy (cBHT) drugs that are currently booming (65). When we refer to HRT in this article, we refer to the body-identical (also called biosimilar) HRT, and not to synthetic HRT or unregulated cBHT. We emphasize the latter distinction due to general confusion with regard to these terms.

Regulated body identical HRT can rightly be considered one of the great achievements of our time, not only with respect to physical problems, but also mental health. This includes mental disorders such as depression, anxiety, and other stress-related disorders; for example, in reproductive transitions like perimenopause, peripartum and the premenstrual transition, particularly in the context of a derailed stress endocrinum, e.g., due to trauma or life stress in earlier life (12). As mental health practitioners, we have witnessed too many cases of middle-aged women in perimenopause, treated with classic psychopharmaceuticals that did not solve cases of depression, anxiety, sleeping disorders, and reduced stress resilience in a satisfactory and permanent manner. Too often, the established psychopharmaceuticals do not provide the desired response with respect to the latter group of psychoneuroendocrine disorders. The treatment with body-identical hormones has been found to solve such mental health problems as well as a whole range of physical (peri-)menopausal problems (66). The important role of 17β-estradiol as the most biologically active estrogen in female cognitive functioning and mood is well-established (67), and the need for psychoendocrinological practices has been discussed to address these types of disorders more specifically (14). In some mental health contexts, arguments for HRT-based treatment are motivated by the long-established origin of a group of mental health problems in steroid hormone imbalances [e.g., as early as (68); later (10, 12); and many others], as well as the medical successes of steroid hormone treatment long seen in practice (10, 69–72). In the meantime, women still make up more than half of the world’s population. In traditional psychiatric institutions where HRT is not yet considered a treatment option for mental health problems, we still witness tragic human fates and enormous healthcare costs over the life span of each of these human beings, who might fall through the cracks of the system. In these cases, psychopharmacological treatments may simply be mis-targeted and potentially lead to the chronic course of mental disorders while still contaminating our water. At the same time, in gynecological practices, psychiatric problems are too often neglected. Similar scenarios may manifest in people with other stress hormone -related disorders such as the so-called “burnout.”

We argue that this psychoneuroendocrine group of disorders could be affected by EDCs, be it via food or drinking water, and that the endocrinological treatment for these disorders could be, in itself, a root cause of the EDC problem, potentially resulting in a vicious circle for humankind. Studies are needed to explore this. The topic of water contamination by steroid hormones will have to form an important part of the highly relevant ongoing discourse and future research. The EU Directive 2001/83/EC was adopted to clarify that the pharmaceutical industry has a role not only in creating, but also in trying to solve the EDC problem, as implemented by the European Medicines Agency (73). The environmental risk assessment (ERA), a scientific process mainly applied in the EU, in particular in the pharmaceutical context, seeks to assess environmental hazards humanity faces by the use of medicinal products. This includes that producers need to evaluate these hazards across the whole lifecycle of the drugs for which they seek marketing authorization. Since its guideline revision in 2024 has been issued, this EU directive has been strengthened by its emphasis of the justification for missing data and is currently evolving toward more transparent, digital processes defined as eERA. Psychoneuroendocrine disorders often fall in between the areas of expertise of health practitioners, a grievance causing people to turn to various forms of unregulated services and products, posing a challenge not only for the human body, but also for different water resources. On the one hand, humanity has evolved to a point at which it faces the hazardous combination of capitalism left unchecked with a high degree of human self-optimization (partly also serving elitist needs and dreams of eternal youth). More than ever before, people are buying unregulated products and services of self-appointed expert practitioners thriving in these gray treatment areas of our time. On the other hand, this is intertwined with a degree of digitalization that allows for a flood of potential misinformation and unregulated products, with consequences for our psychological and physical health, and ultimately, our water. We, as a society, need to reframe the way we address psychoneuroendocrine disorders and their treatment. Even HRT treatment dosing is still somewhat random, with few protections against overdosing, because the subjective well-being of the individual is currently handled as the most relevant benchmark by many practitioners. We therefore also note that mental health medications, be they from the psychiatric or from the endocrinological spectrum, would need to be prescribed in a more targeted and efficiency-controlled manner. In addition to the rational use of medication, alternative treatment concepts with a lower environmental impact should be considered on an individual level. Innovative solutions for reducing pharmaceutical input into the human body, and thereby also into our water, would ideally result in more targeted dosing. This would clearly be a worthwhile goal for further research, with the aim of reducing the burden on the human body as well as the amount of EDCs in water. Furthermore, the use of HRT is expected to increase significantly in the future. Why this development one may ask? Humanity has seen times when the life expectancy of women hardly exceeded menopause and they simply did not live long enough to face the same problems as those faced by women today. Today, women live decades beyond their menopause, having fulfilled often stressful double-roles in their careers as well as at home. At the same time, they wish to enjoy their sexual health as they age, calling for different solutions that include additional health-preserving innovations like HRT.

## 4 How to reduce EDCs: water treatment versus reduction of release

There are two possibilities to deal with EDCs and related effects in the aquatic environment. Either end-of-pipe treatments at wastewater treatment plants or waterworks in order to decrease concentrations of EDCs, or the reduction of EDC emissions into water.

### 4.1 Reducing EDCs by water treatment

Endocrine disrupting compounds represent a heterogenous group of chemicals with different chemical structures and properties. Past studies mainly focused on the removals of prominent individual EDCs (74, 75) or more scarcely on the reduction of endocrine activity based on bio-assays (36, 76). Concentrations of EDCs are often not sufficient to cause detectable endocrine effects in bio-assays (77). While efficient eliminations in conventional wastewater treatment plants or in waterworks are assumed for certain EDCs (78). However, other studies reported insufficient eliminations of several EDCs and endocrine effects in typical treatment processes such as activated sludge processes, aeration, flocculation, and deep-bed filtration (36, 76, 79, 80).

Advanced treatment processes, such as adsorption onto activated carbon or other adsorbents, ozonation, and membrane filtration are able to decrease concentrations of numerous substances (including EDCs) and endocrine effects (36). Review articles of Liu et al. (74) and Werkneh et al. (75) addressed removals of various EDCs (e.g., amitrol, bisphenol A, non-ylphenol, 17α-ethinylestradiol) with different water treatment options.

Adsorption onto granular activated carbon showed effective removal of EDCs, along with the reduction of endocrine effects (36). Prominent phenolic EDCs such as estrone, 17β-estradiol, bisphenol A, and others were also effectively removed by adsorption onto granular or powdered activated carbon or biochar (81–83). As for other target organic micropollutants (84), the adsorption of EDCs depends on the competition with other organic water constituents such as dissolved organic carbon (DOC) (85). Elevated DOC concentrations in wastewater typically reduces the adsorption of EDCs (86). Hydrophobic target compounds with respective functional groups (i.e., phenolic groups) tend to adsorb very well, but predictions are still challenging (87).

Ozonation, a well-researched treatment for the transformation of organic micropollutants (88), shows substantial eliminations of EDCs and endocrine effects (36). Since ozone reacts especially well with double bonds, activated aromatic systems, and non-protonated amines in target molecules (89), EDCs with such moieties are likely transformed in ozonation. Three technical-scale wastewater treatment plants with ozonation showed complete eliminations of estrogenicity and substantial decreases of glucocorticogenic activity (36). Ozonation of drinking water with typical dosages reduced estrogenicity (caused by 17β-estradiol) by a factor of 200, but no complete transformation of 17β-estradiol was achieved, and transformation products were formed (90). Bahr et al. (91) reported efficient elimination of 17α-ethinylestradiol at a specific ozone consumption of 0.5 mg ozone per mg DOC. Advanced oxidation processes such as photocatalysis showed effective oxidation of EDCs (92, 93). Advanced oxidation processes with radical reaction pathways likely eliminate a number of EDCs. For example, Ranjbar et al. (94) observed substantial elimination of BPA by heterogeneous catalysis with persulfate and magnetic graphite intercalation compounds.

Nanofiltration and reverse osmosis showed almost complete removal of 36 EDCs at technical-scale (85) and good results were achieved in the removal of phenolic EDCs at lab-scale (95). An innovative approach for wastewater treatment at lab-scale has been the modification of nanofiltration membranes with graphene oxide, used as nanochannels in membrane polymers. Improved removal effectiveness could be achieved for EDCs due to size exclusion, adsorption, or electrostatic repulsion (96).

Reverse osmosis and nanofiltration treatment produce concentrates as wastewaters, which need to be treated further, whereas during adsorption, EDCs are accumulated on the surface of adsorbents which can be reactivated or energetically utilized after water treatment. Ozonation oxidizes organic micropollutants to transformation products, which might show endocrine effects as well. Granular activated carbon filters as post-treatment after ozonation might be a suitable option, which was also studied to further reduce toxicity due to biotic transformation of micropollutants and their transformation products (97). In general, effectiveness assessments based on the reduction of endocrine effects could directly improve the evaluation of water treatment processes.

### 4.2 Reduction of EDCs in water by reducing EDC emission from the human body: from Wilhelm Waldeyer’s neuron theory to a psychoneuroendocrine system with AI extension

Looking at the immense costs of removing steroid hormones from water, the second option concerning the reduction of EDC release in the first place might constitute a more easily attainable goal. The options for reduction of release known up to now have been thoroughly reviewed by Duh-Leong et al. (98). They point out that interventions and policies at both the individual level and at the level of government can contribute significantly to the reduction of human exposure to EDCs. There is, for instance, scientific evidence that careful choices concerning an organic diet with regulated food packaging, or personal care products and household renovations that avoid EDCs are good examples of how exposure to- and the release of EDCs by human beings can be reduced.

To complement this line of research, our review seeks to focus on a key origin of EDCs in the water cycle: the human body as the EDC emitting unit, however, this time in the context of human health and related excretions, in particular in the context of psychoneuroendocrine disorders and their treatment. Do we have innovations directed at the body itself that assist us in regulating the problem right at its origin: the human body, and in particular the human brain, with the aim of avoiding excessive hormonal treatment and excretion into the water to begin with? We conclude that there is a research gap, also with respect to what can be done in the health sector, providing a key opportunity to develop a solution to the problem of EDCs in our water.

Humanity has come a long way in understanding viable processes of the human body and its functions for health-related issues and general well-being. Functional body parts, some more than others, are also fascinating on its own, especially for example our brain. Understanding its elementary structure is the starting point for two types of challenges, how to understand mental health issues, as well as how to artificially reproduce its function. For both purposes, the further path was paved by Wilhelm von Waldeyer-Hartz with his groundbreaking work on the human nervous system in the 19th century (99). His research on synaptic transmission as a process of chemical and electrical communication in networks responsible for sensory, motor, and cognitive functions provided foundational concepts on the structure and function of the brain, thereby coining the term “neuron” as its basic structural and functional unit. In this work he extensively built on Ramón y Cajal’s groundbreaking microscopic and experimental work and clear rejection of the reticular theory (100), who later received the Nobel Prize together with Camillo Golgi, who made the visualization of individual neurons possible with his Golgi stain (while still championing the older reticular theory). Waldeyer named the new scientific evidence and packaged it in his “neuron theory,” which promoted these new insights to a global platform. Waldeyer stated in his neuron theory that instead of a continuous network fused into one interconnected web with no breaks (reticular theory) nerve cells (neurons) are separate, individual units transmitting signals via nerve fibers to and from one another (99), as well describes how their arrangement influences information processing and transmission in the nervous system (101). Inspired by this depiction of neurophysiological processes and the human nervous system, Warren McCulloch and Walter Pitts developed the first mathematical model of a neural network in 1943 (102). Another milestone was introduced with the first artificial electronic neuron, named the perceptron, developed by Frank Rosenblatt in 1958 (103). These cornerstones are regarded as the basic building blocks of modern artificial neural networks (ANNs). Thus, in current research, Waldeyer’s fundamental concept of the plasticity of the brain is used in the development of adaptive algorithms such as “deep learning,” which can learn from data and adapt (104).

Meanwhile, AI has found diverse applications in medicine, for example in diagnosis and treatment of nervous system disorders. With the help of machine learning, systems can be trained to analyze EEG data such that impending epileptic seizures can be predicted up to 1 h in advance with an accuracy of 99.6%. Applied as an early warning system this can significantly improve safety and quality of life of epileptics (105); in Parkinson’s patients, an AI model is used to predict the response to deep brain stimulation (DBS) to optimally adjust the implanted electrodes in real time (106) Other AI models can detect an increased risk of dementia in Parkinson’s patients at an early stage, based on eye movements (107); rare forms of dementia can be detected by AI models based on MRI images in an early stage. Moreover, other investigations show how recent developments in telemedicine and AI impact the patient-doctor relationship (108).

These isolated examples involve medical practitioners as an intermediary between humans and the underlying computer systems. If medical diagnostics is not the main concern, there is a need to reduce this intermediate translation, for example when information is solely interpreted by end-users for simple day-to-day decisions. For that purpose, data needs to be preprocessed, structured, and coherently visualized.

Virtual Human Twins (VHTs) represent a promising approach to bridge this gap. A VHT is a dynamic, digital representation of an individual that integrates heterogeneous data sources—such as genomics, sensor data, lifestyle information, and clinical records—into a coherent model. These models can simulate physiological processes, predict health outcomes, and provide personalized recommendations. VHTs enable intuitive visualization, real-time simulation, and immediate interpretability and act as a fundament for human enhancement. This is particularly valuable in the context of personalized and preventive medicine by integrating individual variability (109, 110).

Moreover, VHTs can support shared decision-making, enhance patient engagement, and facilitate remote monitoring by making complex health data more accessible and actionable. As such, they are not only tools for simulation but also interfaces for communication between AI systems and human users.

Especially for the EDC topic, such an immediate interface between computer generated information and human beings might be more targeted. Using AI to enhance processes of the microstructure of the human psychoneuroendocrine system, can ultimately be directly related to the hormonal activity in the human body. Analysis of saliva as a diagnostic tool is an example of how this hormonal activity could be accessed. Saliva tests offer a non-invasive and simple method for detecting measurable biomarkers such as hormones, proteins, and metabolic products. Research has achieved relevant progress in this area in recent years. Especially the identification of biomarkers in saliva has led to various health interventions, such as monitoring individual stress levels by measuring cortisol levels in saliva, as a basis for personalized stress management strategies (111); early detection of recurrences in oral and pharyngeal tumors by determining CD44 and total protein levels in saliva to derive appropriate therapeutic measures (112); the analysis of salivary metabolites as a non-invasive method for monitoring glucose metabolism in diabetics (113).

In the following discussion we will summarize and identify gaps in the different scientific disciplines we have reviewed up to this point and will also try to provide an outlook for pathfinders seeking to solve the EDC problem right at the human EDC-emitting body. EDCs constitute a problem for the human nervous system that is at the same time causal to the problem; a vicious circle that Wilhelm Waldeyer couldn’t possibly have foreseen during his pivotal work on the human nervous system in the 19th century. However, the generation of his descendant, his great-great-grandson, our co-author, Götz von Waldeyer-Hartz, will urgently have to deal with in the 21st century. Wilhelm von Waldeyer-Hartz has nonetheless left us a scientific heritage, forming the basis on which AI came into being, which in turn could very well assist us in overcoming this EDC challenge to human health.

## 5 Discussion: EDC reduction by regulating emissions from the human body and at the same time improving human health by a psychoneuroendocrine system with an AI extension?

The interrelated nature of the EDC and human health problems, as well as the need for their respective solutions may have become more obvious in the course of this review. What has also been brought to light is the relationship between the scientific gaps that we identified in the previous sections: Reducing the emission of endocrine-disrupting substances from the human body is an important goal in controlling the damaging effects on ecosystems and people, especially when it comes to dealing with hormonal imbalances (be they related to stress hormones or reproductive hormones or both) in the human body, psyche, and reproductive system. At the same time, AI research is gradually evolving into an innovative solver of a myriad of problems. AI research has neither addressed the challenge of EDCs in water and in the human body, nor explored the problem of untargeted psychoneuroendocrine disorder treatment, and its consequences for the body and this planet’s water. At the same time, endocrinological research using saliva as a biomarker to measure hormones has not yet focused on how hormonal treatments could be more finely adjusted to real-time hormonal status, as might become possible by utilizing AI, thus avoiding excessive hormonal treatments that burden the human organism and our environment.

This review identifies the human body, its disorders-related behaviors, and related health outcomes as one of the key areas where urgent action is needed. Medical use of body-identical HRT, increasingly considered as one of the greater achievements of our time for psychoneuroendocrine health, is currently gaining more popularity, despite its early controversy. Judging from current developments, the use of HRT is expected to become more common in the future, owing to the fact that it is proving to be a very helpful medical intervention, not only in gynecological and psychotherapeutic practice, but also in other disciplines dealing with physical complaints during reproductive transitions, be it perimenstrual, peripartum, perimenopausal, fertility-related or in the context of stress-related disorders. However, scientific evidence still needs to fully catch up with evidence from practical settings.

To safeguard human health from mistargeted pharmacological interventions and to break the circular cycle of EDCs between humans and aquatic ecosystems, innovative strategies are essential. AI-supported extensions of the human psychoneuroendocrine system – enhancing brain plasticity through adaptive learning – could offer a groundbreaking transformative opportunity: reducing the need for psychopharmaceuticals and enabling precise, body-identical hormone therapies. This, in turn, would help minimize the release of excess hormones into water systems, reducing environmental contamination and reabsorption of EDCs by humans through the water cycle. If one were to use biomarkers in saliva as sensory input to an ANN that mimics biological neural networks, then this innovation could be interpreted as a immediate AI-based extension of the human brain.

This could represent another evolutionary step for humans, by improving the efficiency of signal transmission in the nervous system, thus improving human well-being. Would this be seen as part of the evolutionary step from Homo sapiens to “Homo deus,” as it is referred to in other contexts (114)? Alternatively, one could take a more humble look at the human being as moving from that ever more deifying self-image of the great regulator, to the human unit that urgently needs to be regulated itself - be it through the use of hormones, psychopharmaceuticals, weight loss pills etc.,- in fact all of its nature-burdening, unregulated behavior, including that which changes the climate, causing fires and other disasters.

**FIGURE 2:**
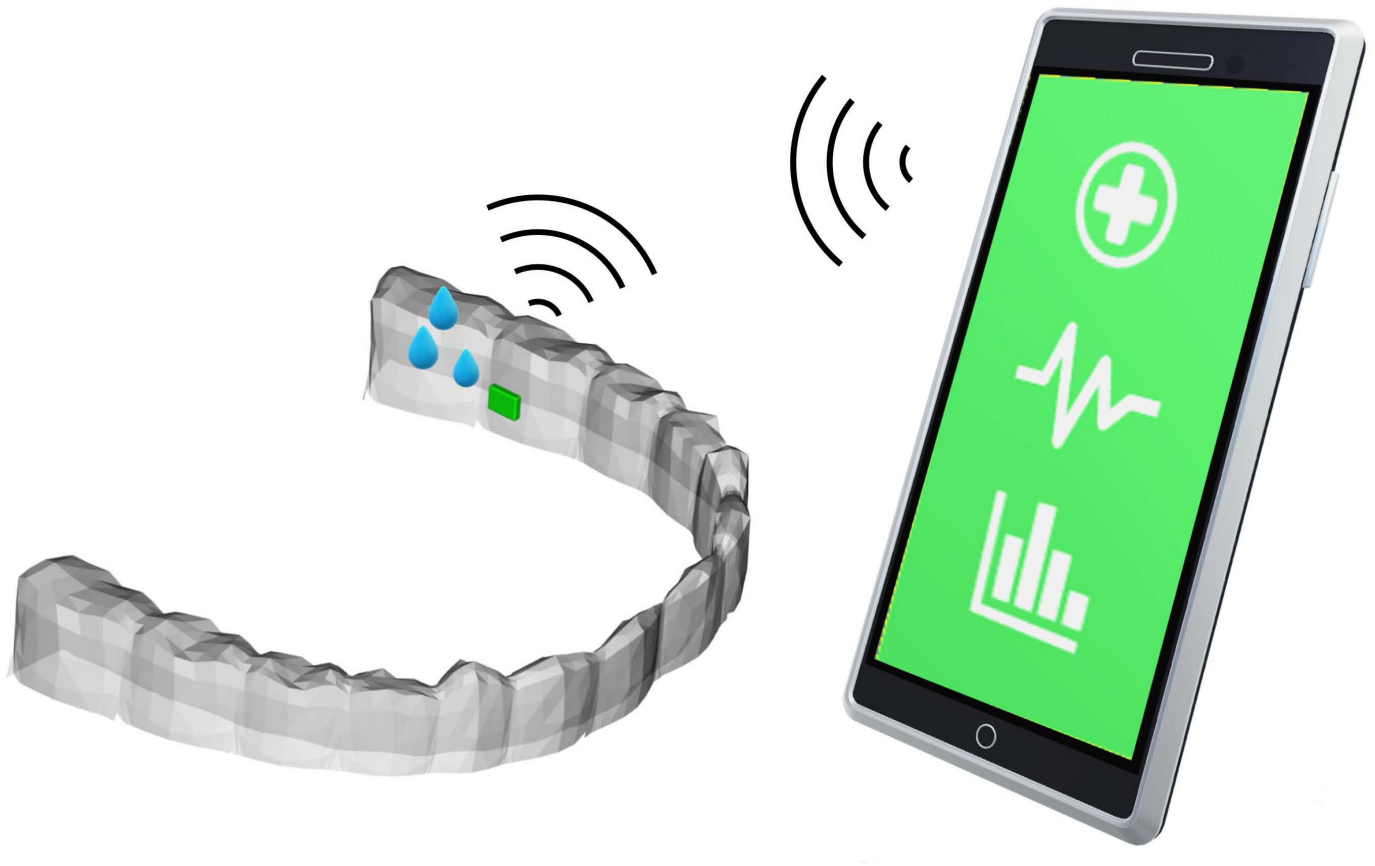
Wearable saliva endocrinological sensor connected to AI-App.

We conclude with a concrete proposal for a solution: An endocrinological sensing wearable, such as a dental night splint usually worn against bruxism/teeth grinding, equipped with a microchip containing a microfluid sensor for continuous hormone measurement via saliva testing on a small scale; A networked wearable device that can connect from the sensor interface to an AI application on a smartphone or smartwatch, incorporating a corrective feedback loop (Figure 2). The data could initially be analyzed by health practitioners and discussed with patients until the psychoneuroendocrine system with the AI synapse extension becomes a self-perpetuating unit. Thus, parallel to academic research that focuses on this border area between AI and psychoneuroendocrinology, there is a need for start-ups that could develop such a dental product, as well as the AI applications for the nocturnal saliva hormone measurement device. Applying such an AI extension to the processes within the microstructure of the psychoneuroendocrine system would mimic biological neural networks using ANNs and provide the potential to establish a medically beneficial corrective feedback loop.

The current state of the art shows that the first portable sensor systems for saliva analysis are being researched, but are not yet widely used (115). Studies focus on miniaturizing and increasing the sensitivity of sensors that can reliably detect biomarkers such as hormones or electrolytes (116). One challenge is to accurately measure low analyte concentrations without interference from other components. Another aspect of the research is energy-efficient data processing. Since continuous saliva analyses generate large amounts of data, powerful algorithms are required. Artificial intelligence could help to identify patterns and also provide personalized health alerts. The combination of saliva sensor technology, AI-supported analysis, and mobile technologies could revolutionize preventative health monitoring. As soon as market-ready systems are available, they could be used not only in clinical diagnostics but also in everyday life (117). Current research shows promising progress in this direction – and we would like to make our contribution.

The benefits are obvious: AI-supported solutions such as the one proposed could identify patterns that correlate with hormonal changes in human beings struggling with psychoneuroendocrine health problems and ultimately correct them. This data-based analysis could lead to a better understanding, prediction, and treatment of symptoms such as mood disorders, anxiety, sleep problems, or other physical complaints. Such treatments could react to changes in real time, assisting in the reduction of hormonal imbalance-related stress, while promoting general well-being through a finely targeted feedback loop and efficiently targeted medication. In the long term, this technology could enable the human species to proactively handle psychoneuroendocrine derailing, and to deepen their understanding of their own well-being and simultaneous protection of their environmental and social environmental. An additional benefit would be the relief of overburdened healthcare systems, where people often have to wait months between appointments. A psychoneuroendocrine system with an AI extension could save enormous costs in national healthcare systems. In the examples of contraception, high-risk pregnancies, or the menopausal transition: birth control pills would no longer be necessary for the whole month; HRT could be effectively reduced to the bare minimum; burdening fertility treatments could be more sensitively balanced, and more. This means reducing unnecessary allostatic load from the human body, which would benefit our environment, as well as the healthcare and economic systems, in which psychoneuroendocrine disorders currently cause enormous costs to be incurred, weakening the performance of the workforce. Going a step further, one could envision science-fiction-like AI extensions to the human psychoneuroendocrine system that could replace the need for drug administration in humans altogether, offering a clean solution to both the human body and the environment.

On the one hand, apart from the pharma industry’s expected adverse reaction to potentially sinking revenues, such visions might invoke dystopic “Brave new world” sentiments in the general population. On the other hand, it may become evident that the vicious circle between pharmacological innovations and the arising problem of EDCs require holistic innovations at all parts of the urban water cycle, including the human body, to keep potential consequences for the planet and its population in check. This is all about creating sustainability in our medical systems. Even in the case that the results of the German Environment Agency in autumn 2025 show only marginal *in vitro* effects of EDCs in drinking water, we should keep in mind the prospect that contraception, infertility treatments, hormone-replacement therapies, pharmaceutical treatments of stress-related disorders and flame retardants etc., are likely to increase in worldwide production in the future; so if the problem seems insignificant now, we will most likely face a much larger one in the future. It is therefore important not only to carry out chemical-based analysis (of individual substances), but also to establish effect-based analysis in order to be able to examine complex (water) samples. In addition, it is of vital importance to focus on the human body as the EDC emitting unit in the urban water cycle and to develop innovations to reduce those emissions. The time has come to prepare for the EDC challenge and to take as many preventative steps as possible.

We need to be aware of the difficulties we will face in digitizing chemical signals of human endocrinology and EDCs for AI-based analysis. Such a process will entail converting highly complex chemical and biological interactions into data that can be read by a machine for pattern recognition, prediction and healthcare providers and policy maker’s decision-making. A very recent review by Huang et al. (118) provides a good overview of these challenges: Chemical signals are complex and the heterogeneity of the data sources will be challenging. Also, high-dimensional chemical descriptors will be needed which can lead to feature explosion and will have to be effective at isolating relevant signals. *In vitro* and *in vivo* differences will have to be taken into account given that biological systems are experimentally noisy posing a major challenge to replicability and signal stability as well as the training of AI models. Another challenge will be non-linear and low-dose effects of EDCs which will make us rethink classical toxicological approaches. The temporal aspect of pulsatile hormone secretion and receptor desensitization will need to be captured by time-series modeling approaches. Other strategies that might help us face these challenges include the use of graph neural networks (GNNs) for molecular structure representation; multi-modal AI architectures that integrate chemical, biological and toxicological data and the implementation of explainable AI (XAI) approaches. Vice versa, another challenge will be the identification of biomarkers in the human body helping us to control any EDC exposure.

We have come a long way; from Waldeyer’s neuron theory to helpful developments like the biopsychosocial model of human health (119), the stress vulnerability model (49), and recently a psychoneuroendocrinological approach to mental health, the path toward visions of a psychoneuroendocrine model with an AI extension is set. We have yet to reach our goal of providing a basis for truly transformational change: This entails completing the complex task of solving the EDC problem while promoting human well-being and simultaneously taking a charitable stance toward the planet we inhabit. This could be the ultimate objective of the interdisciplinary collaborations we aim to inspire through this review. The beneficiaries of such interdisciplinary research initiatives are human beings, our planet, and everything that it sustains.
